# MRI Volumetric Analysis of the Thalamus and Hypothalamus in Amyotrophic Lateral Sclerosis

**DOI:** 10.3389/fnagi.2021.610332

**Published:** 2022-01-03

**Authors:** Shan Ye, Yishan Luo, Pingping Jin, Yajun Wang, Nan Zhang, Gan Zhang, Lu Chen, Lin Shi, Dongsheng Fan

**Affiliations:** ^1^Department of Neurology, Peking University Third Hospital, Beijing, China; ^2^Beijing Municipal Key Laboratory of Biomarker and Translational Research in Neurodegenerative Diseases, Beijing, China; ^3^Brain Research Institute, Shenzhen, China; ^4^Department of Imaging and Interventional Radiology, The Chinese University of Hong Kong, Hong Kong, Hong Kong SAR, China

**Keywords:** amyotrophic lateral sclerosis, thalamus, hypothalamus, volumetric analysis, MRI

## Abstract

**Background:** Increasing evidence has shown that amyotrophic lateral sclerosis (ALS) can result in abnormal energy metabolism and sleep disorders, even before motor dysfunction. Although the hypothalamus and thalamus are important structures in these processes, few ALS studies have reported abnormal MRI structural findings in the hypothalamus and thalamus.

**Purpose:** We aimed to investigate volumetric changes in the thalamus and hypothalamus by using the automatic brain structure volumetry tool AccuBrain^®^.

**Methods:** 3D T1-weighted magnetization-prepared gradient echo imaging (MPRAGE) scans were acquired from 16 patients with ALS with normal cognitive scores and 16 age-, sex- and education-matched healthy controls. Brain tissue and structure volumes were automatically calculated using AccuBrain^®^.

**Results:** There were no significant differences in bilateral thalamic (*F* = 1.31, *p* = 0.287) or hypothalamic volumes (*F* = 1.65, *p* = 0.213) between the ALS and control groups by multivariate analysis of covariance (MANCOVA). Left and right hypothalamic volumes were correlated with whole-brain volume in patients with ALS (*t* = 3.19, *p* = 0.036; *t* = 3.03, *p* = 0.044), while the correlation between age and bilateral thalamic volumes tended to be significant after Bonferroni correction (*t* = 2.76, *p* = 0.068; *t* = 2.83, *p* = 0.06). In the control group, left and right thalamic volumes were correlated with whole-brain volume (*t* = 4.26, *p* = 0.004; *t* = 4.52, *p* = 0.004).

**Conclusion:** Thalamic and hypothalamic volumes did not show differences between patients with normal frontotemporal function ALS and healthy controls, but further studies are still needed.

## Background

Amyotrophic lateral sclerosis is a multiple-system neurodegenerative disease that has a far greater impact than motor system dysfunction. In recent years, more researchers in neurosciences have expanded their interest beyond motor dysfunction and explored other research axes, such as energy homeostasis and sleep disorder, in patients with amyotrophic lateral sclerosis (ALS). Patients with ALS generally have normal or low body mass index (BMI). Weight loss may begin before the onset of motor symptoms, which is probably caused by dysphagia and hypermetabolism. Early weight loss is associated with a higher rate of ALS progression with a grimmer prognosis (Dupuis et al., 2011; Gallo et al., 2013; O’Reilly et al., 2013; Huisman et al., 2015; Ahmed et al., 2016). Clinical trials have demonstrated that strengthening intake may benefit patients with ALS (Wills et al., 2014; Dorst et al., 2015). In addition to energy homeostasis, sleep disorders are also fairly common in patients with ALS (Diaz-Abad et al., 2018; Liu et al., 2018; Congiu et al., 2019). Animal studies have shown that sleep disorders may initiate before the onset of motor symptoms (Liu et al., 2015; Zhang et al., 2018). Interestingly, as both energy metabolism and sleep are closely related to the function of the hypothalamus (Vercruysse et al., 2018), it is not surprising that pathological abnormalities in the hypothalamus were found in patients with ALS (Cykowski et al., 2014; Nakamura et al., 2015). In 33 autopsy-confirmed patients with ALS, six patients presented TDP-43 pathology in the hypothalamus (Cykowski et al., 2014). In the autopsy of a patient with familiar ALS, SOD1 immunohistochemistry showed neuronal cytoplasmic inclusions, glial cytoplasmic inclusions, and dystrophic neurites in the brain and spinal cord, with a predilection for the hypothalamus and central gray matter (Nakamura et al., 2015). Hypothalamic neuropeptide levels were shown to be changed in ALS mouse models (Vercruysse et al., 2016). In addition to these biomolecular abnormalities, structural changes have also been reported: Up to 15% of patients diagnosed with behavioral variant frontotemporal dementia (bvFTD), a similar but different disease within the spectrum of ALS (Strong et al., 2017; van Es et al., 2017), were reported to have hypothalamic atrophy (Bocchetta et al., 2015; Ahmed et al., 2017). However, few studies have reported hypothalamic atrophy in patients with ALS (Gorges et al., 2017).

C9orf72 repeat expansion is one of the most important gene mutations in the ALS-FTD disease spectrum (Gijselinck et al., 2012; Balendra and Isaacs, 2018). In addition to commonly reported brain areas, such as the motor cortex, frontal and temporal lobes, and corpus callosum (Foerster et al., 2013), changes in the thalamus have been the focus of relatively few studies. Machts reported bilateral thalamic atrophy in patients with ALS-FTD (Machts et al., 2015). Schönecker’s study subsequently showed thalamic atrophy in ALS/FTD C9orf72 mutation carriers that extended beyond the expected atrophy in the prefrontal and temporal subregions (Schönecker et al., 2018). However, Westeneng found that, although patients with ALS with C9orf72 repeat expansion had more severe bilateral thalamic atrophy, 21% of patients with ALS without the C9orf72 mutation had a similar neuroimaging phenotype (Westeneng et al., 2016). Chipika also found thalamic atrophy in patients with ALS without the C9orf72 mutation (Chipika et al., 2020). Some earlier studies found structural or functional changes in the thalamus in patients with ALS but lacked cognitive and behavioral assessments (Douaud et al., 2011; Sharma et al., 2011, 2013). Therefore, it remains unclear whether thalamic atrophy could occur in patients with pure ALS.

AccuBrain^®^ (BrainNow Ltd., Shenzhen, China) is a cloud-based National Medical Products Administration (NMPA) and Conformité Européenne (CE)-marked software tool that performs brain MRI segmentation. It is based on a multi-atlas segmentation method with a statistical anatomical atlas previously generated by experienced experts, and it automatically calculates the brain volume of various brain structures. AccuBrain^®^ presents less inter-scanner variability than FreeSurfer and FSL-FIRST based on the comparison of their coefficient of variation values of brain volumetry, especially in basal ganglia structures, such as the thalamus (*p* < 0.001) (Liu et al., 2020). Abrigo’s study showed that AccuBrain^®^ could provide accurate automated hippocampal segmentation in accordance with the EADCADNI standard (Abrigo et al., 2018). The clinical value of AccuBrain^®^ has also been demonstrated in various neurological disease studies (Guo et al., 2019; Wang et al., 2019; Zhao et al., 2019; Dou et al., 2020; Hou et al., 2020; Liu et al., 2020). AccuBrain^®^ may, therefore, be a rapid and sensitive tool for detecting brain structures in neurodegenerative diseases.

The aim of this study was to determine whether thalamic and hypothalamic atrophy occurs in patients with ALS by using the AccuBrain^®^ technique.

## Materials and Methods

### Participants

In total, 16 right-handed patients who met the ALS-revised El Escorial criteria (Brook et al., 2000) at Peking University Third Hospital (PUTH) between 2011 and 2012 and 16 right-handed healthy controls were enrolled. All the patients underwent the Mini-Mental State Examination (MMSE), Frontal Assessment Battery (FAB), verbal fluency (VF), and prospective memory (PM) tests before inclusion. The exclusion criteria were as follows: (1) dementia or cognitive dysfunction, brain trauma, epilepsy, stroke, psychiatric disorders, and other central nervous system diseases; (2) absolute or relative contraindication for MRI; and (3) pregnancy.

The clinical characteristics are shown in Table 1. The patients with ALS were followed up until death; during this follow-up period, contact with eight of 16 patients was lost before this endpoint. The life span was the time from the disease onset to death. All the participants provided informed consent. The study was approved by the ethics committee of PUTH.

**TABLE 1:** Characteristics of the participants: patients with ALS and healthy controls.

Characteristic		ALS patients	Healthy controls	*p*-value
Age (years) (mean ± SD)		51.25 ± 11.19	52.06 ± 11.76	0.86
Education (years) (mean ± SD)		11.75 ± 4.78	12.19 ± 4.83	0.73
Sex	Male	7/16	7/16	1.00
	Female	9/16	9/16	
Onset of disease	Bulbar	3/16		
	Extremities	13/16		
Diagnostic level	Definite	3/16		
	Probable	5/16		
	Lab-supported probable	7/16		
	Possible	1/16		
Disease stage	Advanced ALS	8/16		
	Early ALS	8/16		
Duration of illness (months) (mean ± SD)		14.69 ± 9.16		
Bulbar involvement		12/16		
ALSFRS-R (mean ± SD)		36.75 ± 7.42		
Life span (months) (mean ± SD)		48.25 ± 14.99		
Gene mutation		0/16		

*ALSFRS-R, revised ALS functional rating scale; MMSE, mini-mental state examination. Gene mutation includes C9orf72, SOD1, FUS, and TDP43.*

### MRI Acquisition

The data for 16 patients with ALS and 16 healthy controls were acquired on a Siemens 3.0 T Trio TIM MR scanner. Structural MRI data were obtained using a high-resolution 3-D T1-weighted magnetization-prepared gradient echo imaging (MPRAGE) sequence [192 sagittal slices, no gap, layer thickness = 1 mm; field of view (FOV) = 256 mm × 256 mm; repetition time (TR) = 2,530 ms; echo time (TE) = 3.44 ms; inversion time (TI) = 1,100 ms; acquisition time = 363 s].

### Brain Volumetry

All images were processed using AccuBrain^®^ (BrainNow Medical Technology Ltd., China), which is a brain quantification tool that performs brain structure and tissue segmentation and quantification in a fully automatic mode. Given the T1-weighted MRI data, several brain structures and three major brain tissues are segmented automatically based on prior anatomical knowledge specified by experienced radiologists. The anatomical information is automatically transformed onto the individual brain. The absolute volume (in ml) of 20 brain structures and their bilateral components were automatically calculated. These measures and structures included but were not limited to intracranial volume, whole-brain parenchyma, hippocampus, amygdala, ventricular system, lateral ventricle, third ventricle, inferior lateral ventricle, caudate, putamen, pallidum, hypothalamus, thalamus, pons, midbrain, and gray matter in different lobes.

### Statistics

During enrollment, although trying to meet the similarity between the ALS and control groups in sex, age, and education, paired enrollment was still not strictly performed. Therefore, when comparing the clinical characteristics between the ALS and control groups, the chi-square test was used for sex. For age and education factors, *t*-tests were used when the variables were normally distributed, and Mann–Whitney *U* tests were used when the variables were not normally distributed. Multivariate analysis of covariance (MANCOVA) was used to compare the differences in thalamic and hypothalamic volumes between patients with ALS and controls. During the univariate analysis, when studying the relationship between brain volumes and age, years of education, duration of illness, revised functional rating scale (FRS-R) scores and life span, Pearson correlations were used when the data were continuous, normally distributed and had a linear relationship; otherwise, Spearman correlations were performed. When studying the relationship between brain volumes and sex, bulbar involvement and disease stage, *t*-tests were used when the variables were normally distributed, and Mann–Whitney *U* tests were used when the variables were not normally distributed. ANOVA was used to compare the brain volumes among different diagnostic levels. Then, multivariable linear regression analysis was used. The standard statistical significance level was set at *p* < 0.05. IBM SPSS V20 software was used for all statistical analyses.

## Results

### Comparisons of Thalamic Volume Between Patients With Amyotrophic Lateral Sclerosis and Healthy Controls

Multivariate analysis of covariance was used, in which volumes in the left thalamus and right thalamus were set as two dependent variables; patients with ALS or healthy controls were set as independent variables; and whole-brain volume, age, sex, and education years were set as covariates. The volumes were normally distributed. The patients with ALS and controls were two independent groups. There was homogeneity of variance-covariance matrices assessed using Box’s *M* test of equality of covariance (*F* = 1.249, *p* = 0.290). However, there was no significant difference between the ALS and control groups (*F* = 1.311, *p* = 0.287). Descriptive measures of brain volumes were listed in Supplementary Table 1.

### Comparisons of Hypothalamic Volume Between Patients With Amyotrophic Lateral Sclerosis and Healthy Controls

Multivariate analysis of covariance was used, in which volumes in the left hypothalamus and right hypothalamus were set as two dependent variables; The patients with ALS or healthy controls were set as independent variables; and whole-brain volume, age, sex, and education years were set as covariates. The volumes were normally distributed. The patients with ALS and controls were two independent groups. There was homogeneity of variance-covariance matrices assessed using Box’s *M* test of equality of covariance (*F* = 2.533, *p* = 0.055). However, there was no significant difference between the ALS and control groups (*F* = 1.647, *p* = 0.213). Descriptive measures of brain volumes are listed in Supplementary Table 1.

### Thalamic and Hypothalamic Volumes and Clinical Characteristics

Univariate analysis was first used to choose possible variants for multivariable linear regression analysis. Variants with *p* < 0.1 were chosen (Table 2). Descriptive measures of categorical variables, such as disease stages, sex, and bulbar involvement, are listed in Supplementary Table 2.

**TABLE 2:** Univariate analysis of brain volumes and clinical characteristics.

	Left thalamus	Right thalamus	Left hypothalamus	Right hypothalamus
Age (r, p)	−0.54, 0.03	−0.61, 0.01	0.17, 0.54	0.21, 0.44
Sex (t, p)	0.21, 0.84	0.17, 0.87	1.92, 0.08	1.93, 0.08
Years of education (r, p)	0.45, 0.08	0.41, 0.12	0.27, 0.31	0.37, 0.15
Duration of illness (r, p)	−0.07, 0.79	−0.08, 0.76	−0.20, 0.46	−0.23, 0.39
Bulbar involved (t, p)	0.59, 0.57	0.68, 0.51	0.80, 0.44	0.38, 0.71
Diagnostic level (F, p)	2.54, 0.11	2.07, 0.16	2.30, 0.13	2.46, 0.11
Disease stage (t, p)	0.35, 0.73	0.01, 0.99	2.16, 0.05	2.22, 0.04
ALSFRS-R score (r, p)	−0.07, 0.79	−0.13, 0.64	0.28, 0.29	0.07, 0.79
Life span (r, p)	0.12, 0.78	0.02, 0.95	0.25, 0.55	−0.17, 0.69

*Factors with p < 0.1 were chosen as variants for multiple linear regression.*

Multivariable linear regression analysis was separately performed with bilateral thalamic and hypothalamic volumes as the dependent variables and whole-brain volume and factors chosen above as the independent variables. The results are shown in Tables 3, 4. However, after Bonferroni correction (*p* < 0.0125), left and right hypothalamic volumes were correlated with whole-brain volume in the patients with ALS, while the correlation between age and bilateral thalamic volumes tended to be significant. In the control group, left and right thalamic volumes were correlated with whole-brain volume, and sex was correlated with left hypothalamus volume.

**TABLE 3:** Multiple linear regression of brain volumes and clinical characteristics in ALS.

	Left thalamus	Right thalamus	Left hypothalamus	Right hypothalamus
Regression model (R^2^)	0.63	0.56	0.70	0.66
Regression model (F, p)	6.70, 0.007*	5.11, 0.017*	6.34, 0.007*	5.30, 0.013*
Age (t,p)	2.76, 0.017*	2.83, 0.015*	1.70, 0.117	0.93, 0.374
Whole-brain volume (t, p)	2.21, 0.047*	1.64, 0.127	3.19, 0.009*	3.03, 0.011*
Education year (t, p)	1.12, 0.283	0.69, 0.502	–	–
Sex (t, p)	–	–	0.35, 0.731	0.55, 0.591
Disease stage (t, p)	–	–	0.24, 0.813	0.21, 0.835

Four multiple linear regression models were separately made for patients with ALS, *p < 0.05.

**TABLE 4:** Multiple linear regression of brain volumes and clinical characteristics in controls.

	Left thalamus	Right thalamus	Left hypothalamus	Right hypothalamus
Regression model (R^2^)	0.70	0.73	0.73	0.48
Regression model (F, p)	9.42, 0.002*	10.91, 0.001*	10.60, 0.001*	3.65, 0.045*
Age (t, p)	2.44, 0.031*	2.65, 0.021*	1.05, 0.316	0.67, 0.519
Whole-brain volume (t, p)	4.26, 0.001*	4.52, 0.001*	2.31, 0.040*	1.93, 0.078
Education year (t, p)	0.59, 0.569	0.76, 0.460	–	–
Sex (t, p)	–	–	3.00, 0.011*	1.17, 0.265

Four multiple linear regression models were separately made for controls, *p < 0.05.

### Validation of the Automatic Segmentation of the Thalamus

Validation of the automatic segmentation of the thalamus was conducted by comparing the automatically generated segmentation labels with the corrected labels manually performed based on the automatic results. The dice similarity coefficient (range: 0–1) was used as the metric of segmentation accuracy (Table 5).

**TABLE 5:** Validation of the automatic segmentations of thalamus and hypothalamus.

Brain region	Mean dice	Min-max
Left thalamus	0.998	0.990–1.000
Right thalamus	0.999	0.986–1.000
Left hypothalamus	0.995	0.958–1.000
Right hypothalamus	0.994	0.967–1.000

## Discussion

In this study, hypothalamic volume was not different (*F* = 1.65, *p* = 0.213) between the patients with ALS and healthy controls after adjusting for age, sex, education year, and whole-brain volume. Although relatively few studies have reported hypothalamic volume in patients with ALS, Cykowski and Nakamura found pathological changes in ALS autopsy (Cykowski et al., 2014; Nakamura et al., 2015), and Gorges found hypothalamic atrophy in patients with ALS (Gorges et al., 2017). The probable reasons for the negative result in this study include the following: (1) the study did not include subregional analysis of the hypothalamus, which might have covered some potential significance; (2) the sensitivity of AccuBrain^®^ software: Although the study provided a comparison with post-segmentation manual correction, it was not comparable to validation studies of alternative software (Wolff et al., 2018; Billot et al., 2020); and (3) the sample of this study was small, which might have affected the accuracy of the analyses.

Thalamic volume was also not different (*F* = 1.31, *p* = 0.287) between the patients with ALS and healthy controls after adjusting for age, sex, education year, and whole-brain volume in this study. There were actually more reports of thalamic atrophy than hypothalamic atrophy in the patients with ALS (Douaud et al., 2011; Sharma et al., 2011, 2013; Machts et al., 2015; Westeneng et al., 2016; Christidi et al., 2018; Chipika et al., 2020), although this study did not show a difference. In addition to the probable reasons listed as hypothalamic volume, thalamic atrophy was most commonly reported in the patients with ALS who also had frontotemporal dysfunctions (Machts et al., 2015; Westeneng et al., 2016; Christidi et al., 2018). Other studies did not mention cognitive function (Douaud et al., 2011; Sharma et al., 2011, 2013). Patients with normal cognitive function have seldom been reported. Since the cognition of this group of the patients with ALS was normal, we hypothesize that thalamic atrophy in the patients with ALS may be more related to frontotemporal dysfunction and needs further study.

Age is commonly recognized to have a negative correlation with brain volumes. However, although there were trends of significance between age and thalamic volume, there were no significant differences between age and hypothalamic volume in either the patients with ALS or controls. Gorges’s (Gorges et al., 2017) study found that the age at the onset was correlated with anterior hypothalamic volume but not with total or posterior hypothalamic volumes. Our study did not divide the hypothalamus into anterior and posterior parts; thus, it is unknown whether any subregional differences exist. It is also worth noting that, unlike the thalamus, hypothalamic volume was not correlated with whole-brain volume in healthy controls but was significantly correlated in the patients with ALS. Thus, we speculate that hypothalamic volume may be a potential age-independent biomarker for ALS. Additionally, hypothalamic volume was not correlated with the disease stage. These characteristics suggest that further in-depth investigation is required to determine the structural and functional roles of the hypothalamus.

Generally, there are some limitations of this study. First, the sample size was small, which may have affected the accuracy of the analyses, and the results need confirmation in further large-sample studies. Second, we did not divide subregions of the thalamus and hypothalamus, which may have different brain network connections. Third, we used the AccuBrain^®^ technique to analyze thalamic and hypothalamic volumes. The software is commercial and inaccessible to other researchers; therefore, it is not easy to replicate by fellow colleagues. As our study did not provide commonly accepted measures but only a comparison with the post-segmentation manual correction, it is not comparable to validation studies assessing alternative software. For future work, it will be important to compare volumes by using these different sensitive techniques to confirm the results. Much work remains to be done for automated thalamus and hypothalamus volumetry, requiring larger sample sizes to compensate for the measurement errors and to provide a deeper understanding of the role of the thalamus and hypothalamus.

## Conclusion

Thalamic and hypothalamic volumes did not show differences between the patients with normal frontotemporal function ALS and healthy controls, but further studies are needed.

## Data Availability Statement

The original contributions presented in the study are included in the article/Supplementary Material, further inquiries can be directed to the corresponding author/s.

## Ethics Statement

The studies involving human participants were reviewed and approved by the Ethics Committee of PUTH. The patients/participants provided their written informed consent to participate in this study. Written informed consent was obtained from the individual(s) for the publication of any potentially identifiable images or data included in this article.

## Author Contributions

DF conceived this study and provided financial support. SY, LS, and DF designed the research. SY and LS analyzed the data and wrote the manuscript. PJ, YW, NZ, GZ, and LC collected the data. YL analyzed the data. DF was responsible for project management. All authors contributed to the article and approved the submitted version.

## Conflict of Interest

LS is the director of BrainNow Medical Technology Limited. YL is now employed by BrainNow Medical Technology Limited. The remaining authors declare that the research was conducted in the absence of any commercial or financial relationships that could be construed as a potential conflict of interest.

## Publisher’s Note

All claims expressed in this article are solely those of the authors and do not necessarily represent those of their affiliated organizations, or those of the publisher, the editors and the reviewers. Any product that may be evaluated in this article, or claim that may be made by its manufacturer, is not guaranteed or endorsed by the publisher.
